# Magnitude of central obesity and associated factors among adult patients attending public health facilities in Adama town, Oromia region, Ethiopia, 2022

**DOI:** 10.1186/s41043-023-00397-z

**Published:** 2023-06-17

**Authors:** Mihiret Shawel Getahun, Haji Aman Deybasso, Meyrema Abdo Komicha, Abenet Menene Gurara

**Affiliations:** 1 Public Health Department, Adama General Hospital and Medical College, Adama, Ethiopia; 2 Public Health Department, Adama Hospital Medical College, Adama, Ethiopia; 3 Department of Nursing, Arsi University, Asella, Ethiopia

**Keywords:** Central obesity, Adult patients, Associated factors, Adama town, Ethiopia

## Abstract

**Background:**

Central obesity is excessive accumulation of fat around the abdomen, which is associated with the risk of coronary heart and cerebrovascular diseases. This study determined the magnitude of central obesity among adult patients using the waist-to-hip ratio, which has a superior capacity to measure the risk of developing non-communicable diseases compared to the body mass index used in previous studies in Ethiopia.

**Methods:**

Institutional-based cross-sectional study was conducted among a sample of 480 adults from April 1 to May 30, 2022. A systematic random sampling technique was used to select the study participants. Data were collected by using interviewer-administered structured questionnaires and anthropometric measurements. The data were entered into EPI INFO version 7 and analyzed by Statistical Software for Social Science Version 25. The associations between independent and dependent variables were checked using bivariate and multivariate logistic regression analyses. Adjusted odds ratio and 95% confidence interval were used to measure the strengths of the association. Statistical significance was declared at a *P* value of less than 0.05.

**Results:**

The magnitude of central obesity in this study was 40% (51.2% and 27.4% among females and males, respectively (95% CI 36–44%)). Being a female (AOR = 9.5, 95% CI 5.22–17.9), age range 35–44 (AOR = 7.0, 95% CI 2.9–16.7), 45–64 years (AOR = 10.1, 95% CI4.0–15.2), married (AOR = 2.5, 95% CI 1.3–4.7), high monthly income (AOR = 3.3, 95% CI 1.5–7.3), high consumption of milk and milk products (AOR = 0.3, 95% CI 0.1–0.6), family history of obesity (AOR = 1.8, 95% CI 1.1–3.2) were significantly associated with central obesity among the study participants.

**Conclusion:**

The magnitude of central obesity was higher in the study area. Sex, age, marital status, monthly income, consumption of milk and milk products, and family history of obesity were independent determinants of central obesity. Therefore, it is important to raise awareness about central obesity through behavior change communication that targets the high-risk population.

## Introduction

Globally, there has been a shift in food consumption patterns, and overnutrition has become an emerging serious issue in public health. Problems with overnutrition include overweight, obesity, and non-communicable diseases related to diets [1]. Obesity is an abnormal or excessive accumulation of fat in adipose tissue [2]. It results from an imbalance between energy intake and expenditures [3]. It is a significant public health problem that affects people of all ages and genders in both developing and industrialized nations. It is also connected to number of disorders, such as type 2 diabetes, cardiovascular disease (CVD), metabolic syndrome, hypertension, dyslipidemia, and premature mortality [4–6].

If the condition is left ignored worldwide, an estimated 57.8% of adults will be expected to be classified as obese by 2030 [7]. People who are obese run a higher risk of illness and death. In turn, this leads to a loss of investments in human capital, missed opportunities for economic advancement, and a loss of billions of dollars for medical care [8]. Every year, 1.8 million people die prematurely from non-communicable diseases related to being overweight or obese [9].

Recently, central obesity is emerging as an important driving force behind the deterioration of cardiometabolic risk [10], type 2 diabetes mellitus, and increased secretion of free fatty acids, hyperinsulinemia, insulin resistance, hypertension, and dyslipidemia in the general population [11].

The assessment of abdominal fat is the strongest indicator of visceral fat; thus, International Diabetic Federation (IDF) released a new definition of metabolic syndrome based on anthropometric measurements [12, 13]. Waist circumference (WC), waist-to-height ratio, and waist-to-hip ratio (WHR) are three anthropometric proxies that are frequently used to measure central obesity [14]. Waist-to-hip circumference ratio has a superior capacity to measure central obesity, and the risk of developing non-communicable diseases plays a crucial role in clinical diagnosis compared to the body mass index (BMI) [15].

According to a 2013 study on the global trends in obesity, African adults make up 26.9% of the world's overweight and obese populations [16]. Obesity affects up to 50% of type 2 diabetes patients, with prevalence rates ranging by age and environment: 85% in Tanzania, 83% in Nigeria, and 6.21% in Uganda [17].

Ethiopia is experiencing a serious obesity and overweight epidemic, much like other nations. The percentage of overweight and obese persons has climbed from 3% in 2000 to 8% in 2016 [18]. Recently, the prevalence of obesity was 28.4% in Nekemte town, 39.01% in Bale Zone, and 76.1% in Dire Dawa Towns [19–21]. The primary contributors to the epidemic of central obesity seem to be an increase in sedentary behavior [22] and the substitution of plant-based diets with sugar, animal fats, foods low in starch, dietary fiber, and fruits and vegetables [23].

Previous studies in Ethiopia have attempted to evaluate the magnitude of central obesity using BMI, which is a poor predictor of central obesity. This study aimed to assess the magnitude of central obesity and associated factors among adult patients attending public health facilities in Adama Town in Ethiopia.

## Methods and materials

### Study area and period

Adama town is located in Oromia regional state, East Shewa zone at a distance of 99 km from Addis Ababa. In the town, there are seven health centers, 160 private clinics, four private hospitals, and one governmental Hospital with 70% of health service coverage. The study was conducted among adults attending public health facilities in Adama town from April 1 to May 30, 2022 [24].

### Study design and population

An institution-based cross-sectional study design was conducted from April 1 to May 30, 2022.

### Source populations

The source population was all adult patients attending the adult outpatient department of (OPDs) Adama public health facilities during the study period.

### Study populations

The study population was adult patients attending the adult OPDs of randomly selected public health facilities in Adama public health facilities during the study period.

### Inclusion and exclusion criteria

#### Inclusion criteria

Adults aged 18–64 years were included.

#### Exclusion criteria

Pregnant women and individuals who had a physical deformity in any form to the point that obtaining anthropometric measurements were excluded from the study.

### Sample size and sampling techniques

#### Sample size determination

The sample size was determined using the single population proportion formula by considering a 95% confidence interval and a 28.4% prevalence of central obesity in Nekemte Town [19].

Again, a margin error of 4% was calculated from the aforementioned study to boost the power of the survey's findings. Thus, 464 was the estimated sample size for this study. The ultimate sample size was 516 patients after accounting for a 10% non-response rate.

Epi Info version 7, the StatCalc formula was used to calculate the sample size for the second specific objective under the following assumptions: 80% power, 95% confidence intervals, and the independent components of central obesity displayed below.

**Table:** 

Factors	CI (%)	Power (%)	Adjusted odds ratio	% in the unexposed group (%)	% in the exposed group (%)	Sample size	10% non-response rate	Final sample size (*n*)	Refs.
Physical inactivity	95	80	0.35	39.2	18.5	344	35	**379**	[25]
Age > 50	95	80	7.2	27	73	86	9	**95**	[9]
Female sex	95	80	9.3	4	28	174	18	**192**	[26]

#### Sampling procedure and technique

From among the existing eight public health institutions, four were first chosen at random using a lottery technique (Adama Hospital Medical College, Adama Health Centre, Biftu, and Geda Health Centres). The computed sample size was then proportionally assigned to each health facility based on the average daily patient flow over a period of three consecutive months as established by referring to the registration log book. After K was determined, the sample was proportionally divided, and the study subjects were then chosen using systematic random sampling. Hence, *K* = *N*/*n* (949/516) ≈2 where *N* is the total number of adults attending the adult OPDs during the data collection period and *n* is the total sample size. Among the first two patients, the first patient to be interviewed was chosen by lottery. Finally, based on their order of arrival, every second adult patient was recruited.

### Dependent variable

$${\text{Central obesity}} = {\text{yes}}/{\text{no}}$$

### Independent variable

#### Sociodemographic factors

- *Behavioral factors* Smoking, drinking alcohol, physical inactivity.
- *Dietary factors* Consumption of fruits and vegetables, eating out of the home, snacking, fast food consumption, food frequency.
- *Individual factors* knowledge about obesity, and family history of obesity.

### Operational definitions

Fast food intake is defined as eating fried foods, such as burgers, pizza, chips, and biscuits, at least once a week. Participants who indicated they had consumed at least one standard alcoholic beverage during the reporting periods were considered to be alcohol drinkers. Current smokers were defined as respondents who had smoked 100 cigarettes or more in their lives and did so at the time of the survey. Finally, individuals were categorized as having low knowledge or a high understanding of obesity based on the mean of 12 standard questions [19, 27].

### Data collection procedures and instruments

Data were collected by two male and two female BSc nurses. Male nurses collected information from male patients, while female nurses collected data from female patients. The data gathering techniques were supervised by two qualified health professionals with master's degrees. An interviewer-administered semi-structured questionnaire, which was adapted from WHO-STEP wise for non-communicable diseases risk surveillance [28], and FAO [27] were used to collect data. Anthropometric measurements were taken after the interview. First, the questionnaire was prepared in the English language and was translated to Afaan Oromo and Amharic languages and then translated back to the English language to keep the consistency of the tool. The instrument consists of socio-demographic, behavioral, diet-related, physical activity questions, knowledge about obesity, and anthropometric measurements of waist and hip circumference.

The food frequency questionnaire (FFQ) was adapted from the WHO-STEP wise approach to assessing the dietary habits of the study population. The FFQ consisted of seven food groups with a frequency response section, for subjects to report how often each item was consumed over a specified period. The respondents were asked how often they ate the food items with a response category of < 1 time a month, 1–2 times a month, 3–4 times a month, 5–6 times a month, 1 time a day, 2–3 times a day, 4–5 times and ≥ 6 times a day. In addition to FFQ, eating habit (meal pattern) was assessed using an eating habit questionnaire [28].

The questionnaire developed by WHO for physical activity surveillance was used to assess the physical activity pattern among selected participants which is based on the intensity, duration, and frequency of physical activity at work, in recreational settings, and involving transportation (journeys) using a set of 16 questions [28]. Data were collected on the number of days, hours, and minutes of physical activity performed at work, involving transportation, and in recreational settings for at least 10 min or more continuously each day.

To assess the knowledge of the participants about obesity, twelve standard questions adopted from FAO were used [27]. Based on these questions, the mean of their score was taken to classify them as having poor knowledge or good knowledge of obesity.

Waist circumference was measured at the midpoint between the lower margin of the least palpable rib and the top of the anterior superior iliac crest in the mid-axillary line using non-stretchable fixed tension tape wrapped around at this point, parallel to the floor, ensuring it is adjusted without compressing the skin. While taking the measurement, the participant wearing light clothing stood relaxed with feet close together, arms at the side, and body weight evenly distributed. The measurements were taken at the end of a normal expiration. Each measurement was repeated twice; when the measurements are within 1 cm of one another, the average was calculated. When the difference between the two measurements exceeded 1 cm, the two measurements were repeated. The hip circumference was measured at a level parallel to the floor and at the largest circumference of the buttocks. Both measurements were taken with a stretch-resistant tape meter that was wrapped snugly around the subject, but not to the point that the tape was constricting. The tape was kept leveled and parallel to the floor at the point of measurement [28].

### Data quality control

Data quality was ensured starting from tool development to analysis. The training was given to the data collectors and supervisors on sampling procedures, techniques of interview, and the data collection process by the principal investigator for two days, and any doubt in the questionnaire was clarified. Demonstration of measurement was performed for each data collector, on volunteer adults.

A pretest was conducted on 5% of the total sample size (26 adults) attending Hawas health center, which was not selected in the study before the actual data collection to check for clarity and understandability of the questionnaire. Those questions that were found to be unclear or confusing were modified accordingly.

During data collection completeness and logical consistency, checks were made in the setting with close day-to-day supervision to ensure the appropriateness of anthropometric measurements. Data coding, entry, and cleaning were performed by the principal investigator, and after data collection, the supervisor and the PI together recheck the completeness and consistency of the questionnaire where a non-overlapping numerical code was assigned for each question.

### Data processing and analysis

Data were coded, entered, and cleaned by Epi info version 7 and then exported into SPSS version 21 for statistical test analysis. The level of physical activity of the study participants was analyzed using WHO physical activity analysis guidelines. To analyze dietary patterns, the frequency of each food group was converted into tertiles: the highest tertile was used to define ‘‘high’’, the middle tertile was used to define moderate and lower tertiles have been labeled as ‘‘low’’. Normality for continuous variables was checked using the Shapiro–Wilk test. Descriptive statistical analysis was conducted using frequency, percentage, mean, median, interquartile range (IQR), and standard deviation (SD). To identify factors associated with central obesity, both bivariable and multivariable binary logistic regression analyses were done, and the variables in bivariable analysis with *p* values < 0.25 were fitted to the final multivariable logistic regression to adjust for potential confounders to identify the determinants of central obesity among adults. Hosmer and Lemeshow’s goodness of fit test was employed to check the model fitness considering good fit at *p* value ≥ 0.05. Multi-collinearity was checked using variation inflation factor (VIF) at VIF > 10 indicating the presence of multi-collinearity. In the final model, variables with a *P* value < 0.05 were considered statistically significant and an AOR of 95% CI was used to determine the strength of the association. Finally, the results were interpreted and displayed using texts, tables, and figures.

## Results

### Sociodemographic and economic characteristics

A total of 480 patients participated in this study making a response rate of 93.02%. Of the total respondents, 254 (52.9%) were females. The mean (± SD) age of participants was 30 (± 11.5) years. One hundred and seventy-four (36.3%) of them were between 25 and 34 years old. Regarding their religion, 225 (46.9%) were Orthodox Christians. Among the total, 301 (62.7%) were married, and 163 (33.9%) attended college and above. The median (IQR) monthly income of the respondents was 2700 (1500–4000) ETB (Table 1).

**Table 1:** Sociodemographic and economic status of the respondents in Adama town, Oromia, Ethiopia, 2022 (*n *= 480)

Variable	Frequency	Percent %
*Sex*		
Male	226	47.1
Female	254	52.9
*Age in years*		
18–24	112	23.3
25–34	174	36.3
35–44	98	20.4
45–64	96	20
*Religion*		
Orthodox	225	46.9
Muslim	185	38.5
Catholic	8	1.7
Protestant	54	11.3
Others*	8	1.6
*Marital status*		
Never married	154	32.1
Separated/divorced	25	5.2
Married	301	62.7
*Ethnicity*		
Oromo	313	65.2
Amhara	110	22.9
Gurage	37	7.7
Other**	20	4.2
*Educational status*		
No formal education	50	10.4
Elementary (1–8)	114	23.8
Secondary (9–12)	153	31.9
College and above	163	33.9
*Total monthly income*		
< 1500	119	24.8
1500–2699	118	24.6
2700–3999	83	17.3
≥ 4000	160	33.3
*Family size*		
1	22	4.6
2–4	202	42.1
≥ 5	256	53.3

*were Waaqeffanna

**were Tigre, Wolaita

### Behavioral characteristics of the study subject

Twenty-four (5%) reported a history of smoking cigarettes of which (2.5%) were current smokers. Of the participants, 64 (13.3%) drank alcohol within the last month. Of the total, 411 (85.6%) of the participants had a sleep duration of 6–10 h. Regarding physical activity, 354 (73.8%) of them were physically active (Table 2).

**Table 2:** Behavioral and related factors of the respondents in Adama town, Oromia, Ethiopia, 2022 (*n *= 480)

Variable	Frequency	Percent %
*Smoking status*		
Never smoker	456	95
Current smoker	12	2.5
Former smoker	12	2.5
*Drink alcohol last month*		
No	416	86.7
Yes	64	13.3
*Drunk alcohol within 12 months*		
No	403	84
Yes	77	16
*Heavy drinkers*		
No	418	87.1
Yes	62	12.9
*Sleep duration*		
6–10 h	411	85.6
< 6 h	64	13.3
≥ 10 h	5	1.1
*Physical activity*		
Physically active	354	73.8
Physically inactive	126	26.2

### Dietary practices of the respondents

Of the total respondents, 327 (68.1%) of the respondents had eaten a snack, and 320 (66.7%) of the study participants had eaten meals outside of the home. The dietary patterns of the participants indicated that 238 (49.6%) were in the lowest tertiles of fruit and vegetable consumption. Based on the data from food consumption frequency, 158 (32.9%) of the participants had a high consumption of cereal-based staple foods, while 166 (34.6%) had a high consumption of legumes. Of the total, 155 (32.3%) of respondents were in the highest tertiles of milk and milk products consumption, and 153 (31.9%) were in the highest tertiles of sweet and sweetened beverages consumption (Table 3).

**Table 3:** Dietary practices of the respondents in Adama town, Oromia, Ethiopia, 2022 (*n *= 480)

Variable	Frequency	Percent %
*Snack consumption*		
Yes	327	68.1
No	153	31.9
*Fast food consumption*		
Never	348	72.5
1–2 days/week	101	21
≥ 3 days/week	31	6.5
*Eat out of the home*		
Yes	320	66.7
No	160	33.3
*Grain consumption*		
Low	168	35
Moderate	154	32.1
High	158	32.9
*Legumes consumption*		
Low	220	45.8
Moderate	94	19.6
High	166	34.6
*Fruit and vegetable consumption*		
Low	238	49.6
Moderate	138	28.8
High	104	21.6
*Meat, egg, and fish consumption*		
Low	104	21.7
Moderate	246	51.2
High	130	27.1
*Milk and milk product consumption*		
Low	141	29.4
Moderate	184	38.3
High	155	32.3
*Oil and Butter*		
Low	92	19.2
Moderate	250	52.1
High	138	28.7
*Consumption of Sweetened beverage*		
Low	146	30.4
Moderate	181	37.7
High	153	31.9

### Individual factors

Concerning the family history of obesity, 182 (71.7%) respondents had no family history of obesity, whereas 285 (59.4) of the respondents had good knowledge about central obesity.

#### The magnitude of central obesity

**Fig. 1:**
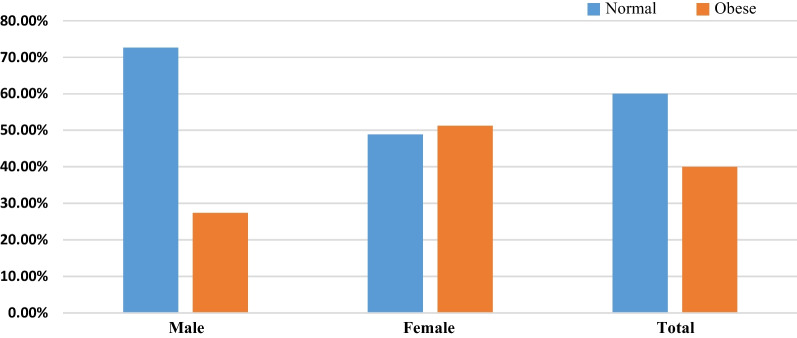
Magnitude of central obesity by sex among adults attending public health facilities in Adama town, central Ethiopia, 2022 (*n *= 480)

The overall magnitude of central obesity was 40% (95% CI 0.36–0.44). In a separate analysis, it was 51.2% (95% CI 0.45–0.57) among females and 27.4% (95% CI 0.22–0.33) among male adult patients (Fig. 1).

#### Factors associated with central obesity

In bivariate analysis variables like age, sex, marital status, educational level, monthly income, sleep duration, physical inactivity, consumption of fruit and vegetables, consumption of meat, egg, and fish, consumption of milk and milk products, family history of obesity, low consumption of fruits and consumption of fast food had a *p* value of < 0.25 and selected at as candidate variables for multivariable logistic regression analysis.

In multivariable analysis, sex, age, monthly income, marital status, consumption of milk and milk products, and family history of obesity persisted to be statistically significant variables associated with central obesity at a *p* value of < 0.05.

Hence, the odds of central obesity were 9.5 times higher among female adult patients than males (AOR = 9.5, 95% CI 5.2–17.9). Besides, the odds of developing central obesity were 7 (AOR = 7.0, 95% CI 2.9–16.7) and 10.1 (AOR = 10.1, 95% CI 4.0–15.2) times higher among individuals in the age group of 35–44 and 45–64 years as compared to those age group of 18–24, respectively. Respondents having monthly income ≥ 4000 Birr had 3.3 times higher odds of central obesity as compared to those having < 1500 ETB monthly income (AOR = 3.3, 95% CI 1.5–7.3). Married adult patients had 2.5 times higher odds of central obesity (AOR = 2.5, 95% CI 1.3–4.7) than those who were never married. Respondents having a family history of obesity had 1.8 higher odds of central obesity as compared to their counterparts (AOR = 1.8, 95% CI 1.1–3.2). On the other hand, the odds of central obesity were 70% lesser among individuals with high consumption of milk and milk products as compared to those with low consumption of milk and milk products (AOR = 0.3, 95% CI 0.1–0.6) (Table 4).

**Table 4:** Factors associated with central obesity among adults attending public health facilities in Adama Town, Oromia, Ethiopia, 2020

Characteristics	Central obesity		COR (95%CI)	AOR (95%CI)
	Yes	No		
	*n* (%)	*n* (%)		
*Sex*				
Male	62 (27.4)	164 (72.6)	1	1
Female	130 (51.2%	124 (48.8)	2.7 (1.8–4.0) *	9.5 (5.2–17.9) **
*Age in years*				
18–24	22 (19.6)	90 (80.4)	1	1
25–34	52 (29.9)	122 (70.1)	1.7 (0.9–3.0)	1.7 (0.8–3.6)
35–44	58 (59(59.2)	40 (40.8)	5.9 (3.2–10.9) *	7.0 (2.9–16.7) **
45–64	60 (62.5)	36 (37.5)	6.8 (3.6–12.7) *	10.1 (4.0–15.2) **
*Marital status*				
Never married	32 (20.8)	122 (79.2)	1	1
Separated/divorced	16 (64)	9 (36)	7.0 (2.7–16.7) *	2.3 (0.7–7.1)
Married	144 (47.8)	157 (52.2)	3.4 (2.2–5.4) *	2.5 (1.3–4.7) **
*Educational status*				
College and above	71 (43.6)	92 (56.4)	1	1
Secondary (9–12)	64 (41.8)	89 (58.2)	0.9 (0.5–1.4)	0.9 (0.5–1.8)
Primary (1–8)	36 (31.6)	78 (68.4)	0.5 (0.3–0.9) *	0.4 (0.2–1.8)
No formal education	21 (42)	29 (58)	0.9 (0.4–1.7)	0.2 (0.1–1.5)
*Total monthly income*				
< 1500	37 (31.1)	82 (68.9)	1	1
1500–2699	42 (35.6)	76 (64.4)	1.2 (0.7–2.1)	0.8 (0.4–1.6)
2700–3999	34 (41)	49 (59)	1.5 (0.8–2.7)	1.7 (0.7–3.7)
≥ 4000	79 (49.4)	81 (50.6)	2.1 (1.3–3.5) *	3.3 (1.5–7.3) **
*Physical activity*				
Physically active	126 (35.6)	228 (64.4)	1	1
Physically inactive	66 (52.4)	60 (47.6)	1.2 (1.3–3.0) *	1.5 (0.8–2.5)
*Parent obesity*				
No	124 (36.4)	217 (63.6)	1	1
Yes	68 (48.9)	71 (51.1)	1.6 (1.1–2.4) *	1.8 (1.1–3.2) **
*Fruit and vegetable consumption*				
High	32 (30.8)	72 (69.2)	1	1
Moderate	62 (44.9)	76 (55.1)	1.8 (1.1–3.1) *	2.0 (1.0–3.9)
Low	98 (41.2)	140 (58.8)	1.5 (0.9–2.5)	1.6 (0.9–3.0)
*Milk and milk product consumption*				
Low	60 (42.6)	81 (57.4)	1	1
Moderate	81 (44)	103 (56)	1.0 (0.6–1.6)	0.7 (0.4–1.3)
High	51 (32.9)	104 (67.1)	0.6 (0.4–0.9) *	0.3 (0.1–0.6) **
*Meat, egg, and fish consumption*				
High	39 (37.5)	65 (62.5)	1	1
Moderate	90 (36.6)	156 (63.4)	0.9 (0.5–1.5)	1.1 (0.6–2.2)
Low	63 (48.5)	67 (51.5)	1.5 (1.1–2.6) *	1.6 (0.8–3.5)
*Consumption of fruit in a day*				
High	14 (70)	6 (30)	1	1
Low	178 (38.7)	282 (61.3)	0.27 (0.1–0.7) *	0.2 (0.05–1.7)
*Consumption of fast food*				
Never	140 (40.2)	208 (59.8)	1	1
1–2 days/week	43 (42.6)	58 (57.4)	1.1 (0.7–1.7)	1.5 (0.8–2.9)
≥ 3 days/week	9 (29)	22 (71)	0.6 (0.2–0.9) *	0.6 (0.2–1.9)

*Significant at *p* value < 0.25 in unadjusted logistic regression analysis

**Significant at *p* < 0.05 in adjusted logistic regression analysis

## Discussion

The current cross-sectional study was conducted to assess the magnitude of central obesity and associated factors among adults attending public health facilities in Adama town. The overall magnitude of central obesity among adults attending public health facilities in Adama town was 40% (95% CI 36–44%). The result reported in this study is comparable with the findings from the studies done in Gonder, and Dabat town (37.6%), South East Ethiopia (39%), central Tanzania (41.8%), India (46.6%) [20, 29–31], but higher than the studies conducted in Nekemte town, West Ethiopia (28.4%) [19], Woldia town, Northeast Ethiopia (16.5%) [26], Dilla town South Ethiopia (24.4, %) [25] and Burkina Faso (22.5%) [14]. Differences in the cutoff value for waist circumferences could be a possible reason for variations in the magnitude of abdominal obesity between studies [14, 25]. Moreover, discrepancies in the findings could be explained by differences in the age distributions [14, 19].

On the other hand, our finding is lower than study findings done in Dire Dawa city, Eastern Ethiopia (76.1%) [21], Eastern Sudan (67.8%) [5], South Africa (67%) [32], and Portugal (50.5%) [33]. The discrepancies in the study results may be attributed to methodological differences, the setup in which the study was conducted, variations in the cutoff criteria, and differences in body composition and shape across ethnic groups [34].

In this study, female adults had higher odds of central obesity compared to men. This finding supports studies done in different parts of Ethiopia [25, 26, 30] and studies in South Africa and China [32, 35]. The precise biochemical connection between being female and central obesity has not yet been elucidated. However, the association between being female and central obesity can be explained by the fact that females usually have higher fat distribution than males, particularly with a larger area of subcutaneous adipose tissue in the abdomen [36]. Moreover, women who live in developing nations, such as Ethiopia, stay at home and engage in less physical activity that requires lower energy expenditure than men [37].

In this study, the odds of abdominal obesity increased with age, a finding that is consistent with the study done in Tanzania, Ivory Coast, and China [9, 35, 38, 39]. The relationship is conceivable, given that older persons gradually have slower metabolic rates, engage in fewer physical activities, and lead more sedentary lifestyles. Furthermore, older people have a higher risk of developing central obesity due to the change in the distribution and accumulation of abdominal fat among older people [38].

In this study, there was a statistically significant association between marital status and increased risk of central obesity. Hence, married participants had higher odds of being centrally obese as compared to those who were never married. Similar findings were reported from the studies done in Uganda, South Africa, and Argentina [32, 39, 40]. The reasons could be due to a potential shift in dietary patterns since married persons are more likely to have consistent eating habits and social support resulting from the duty of dining together [41].

The odds of central obesity were higher for individuals who had a higher average monthly income than those with a lower monthly income. The finding is in line with a study done on Indian adults and another study in Indonesia [42, 43]. Individuals who earn higher incomes may alter their dietary habits and favor high-energy meals. In addition, a higher monthly income can result in more people using motorized modes of transportation. Instead, those with lower incomes might be more active and eat low-energy, high-fiber foods, which are less popular and more affordable in developing countries [44].

In this study, the odds of central obesity were lesser for individuals with high consumption of milk and milk products as compared to those with low consumption. This finding is supported by studies done in Sweden, the USA, and India [45–47]. The association has been hypothesized that calcium in dairy foods lower levels of body fat through its effects on lipogenesis [48]. Milk is also an important source of protein and protein-rich diets that have been shown to promote satiety and a rich source of the essential amino acid leucine that is involved in the partitioning of dietary energy [48].

This study also found that the odds of being centrally obese were higher for adult patients with a family history of obesity as compared to those who had no family history of obesity. This is alike to the study findings in southwest Ethiopia, Iran, and Italy [49–51]. Though the exact mechanism connecting the family history with the occurrence of obesity is not completely understood, it could be influenced by genetic, environmental factors and shared family lifestyle characteristics.

### Limitations of the study

The study's main limitation is the nature of the cross-sectional study design, which makes it challenging to ascertain the temporal relationships. Furthermore, the frequency of dietary practices, physical activity, and behavioral factors may be influenced by recall and social desirability bias.

## Conclusion

The magnitude of central obesity in this study was higher compared to previous studies. Being a female, age group of 35–44, and 45–64 years, being married, high monthly income, high consumption of milk and milk products, family history of obesity were the predictors of central obesity based on WHR in this study of adult patients attending public health facilities in Adama town.

It is crucial to develop routine obesity screening procedures. Governmental and non-governmental organizations should collaborate with other stakeholders to offer behavioral change communication programs that target high-risk populations. Future studies are required to look into how dairy products affect weight gain and abdominal obesity.

## Data Availability

The data that support the findings of this study are available from the corresponding author upon reasonable request.

## Abbreviations

AOR: Adjusted odds ratio
BMI: Body mass index
CVD: Cardiovascular disease
FAO: Food and Agriculture Organization
IDF: The International Diabetic Federation
IQR: Interquartile range
IRB: Institutional Review Board
OPD: Outpatient department
SD: Standard deviation (SD).
SPSS: Statistical Package for the Social Science
USA: United States of America
WC: Waist circumference
WHO: World Health Organization
WHR: Waist-to-hip ratio
WR: Waist-to-height ratio
