# Correction to: Magnitude of central obesity and associated factors among adult patients attending public health facilities in Adama town, Oromia region, Ethiopia, 2022

**DOI:** 10.1186/s41043-023-00457-4

**Published:** 2023-10-19

**Authors:** Mihiret Shawel Getahun, Haji Aman Deybasso, Meyrema Abdo Komicha, Abenet Menene Gurara

**Affiliations:** 1 Public Health Department, Adama General Hospital and Medical College, Adama, Ethiopia; 2 Public Health Department, Adama Hospital Medical College, Adama, Ethiopia; 3 Department of Nursing, Arsi University, Asella, Ethiopia

**Correction to: Journal of Health, Population and Nutrition (2023) 42:57** 10.1186/s41043-023-00397-z

Following publication of the original article [1], the authors identified an error in Table 4. The correct table is given below.

**Table 4:** Factors associated with central obesity among adults attending public health facilities in Adama Town, Oromia, Ethiopia, 2020

Characteristics	Central obesity		COR (95%CI)	AOR (95%CI)
	Yes	No		
	*n* (%)	*n* (%)		
*Sex*				
Male	62 (27.4)	164 (72.6)	1	1
Female	130 (51.2%	124 (48.8)	2.7 (1.8–4.0) *	9.5 (5.2–17.9) **
*Age in years*				
18–24	22 (19.6)	90 (80.4)	1	1
25–34	52 (29.9)	122 (70.1)	1.7 (0.9–3.0)	1.7 (0.8–3.6)
35–44	58 (59(59.2)	40 (40.8)	5.9 (3.2–10.9) *	7.0 (2.9–16.7) **
45–64	60 (62.5)	36 (37.5)	6.8 (3.6–12.7) *	10.1 (4.0–15.2) **
*Marital status*				
Never married	32 (20.8)	122 (79.2)	1	1
Separated/divorced	16 (64)	9 (36)	7.0 (2.7–16.7) *	2.3 (0.7–7.1)
Married	144 (47.8)	157 (52.2)	3.4 (2.2–5.4) *	2.5 (1.3–4.7) **
*Educational status*				
College and above	71 (43.6)	92 (56.4)	1	1
Secondary (9–12)	64 (41.8)	89 (58.2)	0.9 (0.5–1.4)	0.9 (0.5–1.8)
Primary (1–8)	36 (31.6)	78 (68.4)	0.5 (0.3–0.9) *	0.4 (0.2–1.8)
No formal education	21 (42)	29 (58)	0.9 (0.4–1.7)	0.2 (0.1–1.5)
*Total monthly income*				
< 1500	37 (31.1)	82 (68.9)	1	1
1500–2699	42 (35.6)	76 (64.4)	1.2 (0.7–2.1)	0.8 (0.4–1.6)
2700–3999	34 (41)	49 (59)	1.5 (0.8–2.7)	1.7 (0.7–3.7)
≥ 4000	79 (49.4)	81 (50.6)	2.1 (1.3–3.5) *	3.3 (1.5–7.3) **
*Physical activity*				
Physically active	126 (35.6)	228 (64.4)	1	1
Physically inactive	66 (52.4)	60 (47.6)	1.2 (1.3–3.0) *	1.5 (0.8–2.5)
*Parent obesity*				
No	124 (36.4)	217 (63.6)	1	1
Yes	68 (48.9)	71 (51.1)	1.6 (1.1–2.4) *	1.8 (1.1–3.2) **
*Fruit and vegetable consumption*				
High	32 (30.8)	72 (69.2)	1	1
Moderate	62 (44.9)	76 (55.1)	1.8 (1.1–3.1) *	2.0 (1.0–3.9)
Low	98 (41.2)	140 (58.8)	1.5 (0.9–2.5)	1.6 (0.9–3.0)
*Milk and milk product consumption*				
Low	60 (42.6)	81 (57.4)	1	1
Moderate	81 (44)	103 (56)	1.0 (0.6–1.6)	0.7 (0.4–1.3)
High	51 (32.9)	104 (67.1)	0.6 (0.4–0.9) *	0.3 (0.1–0.6) **
*Meat, egg, and fish consumption*				
High	39 (37.5)	65 (62.5)	1	1
Moderate	90 (36.6)	156 (63.4)	0.9 (0.5–1.5)	1.1 (0.6–2.2)
Low	63 (48.5)	67 (51.5)	1.5 (1.1–2.6) *	1.6 (0.8–3.5)
*Consumption of fruit in a day*				
High	14 (70)	6 (30)	1	1
Low	178 (38.7)	282 (61.3)	0.27 (0.1–0.7) *	0.2 (0.05–1.7)
*Consumption of fast food*				
Never	140 (40.2)	208 (59.8)	1	1
1–2 days/week	43 (42.6)	58 (57.4)	1.1 (0.7–1.7)	1.5 (0.8–2.9)
≥ 3 days/week	9 (29)	22 (71)	0.6 (0.2–0.9) *	0.6 (0.2–1.9)

*Significant at *p* value < 0.25 in unadjusted logistic regression analysis

**Significant at *p* < 0.05 in adjusted logistic regression analysis

Table 4 has been updated in this correction and the original article [1] has been corrected.
